# Diagnostic performance of liver steatosis analysis and ultrasound-guided attenuation parameter in quantifying hepatic steatosis: a comparative evaluation using controlled attenuation parameter as reference

**DOI:** 10.3389/fphys.2026.1752895

**Published:** 2026-02-27

**Authors:** Juan Chen, Yanhong Hao, Zhilong Liu, Lele Song, Ping Liang, Liping Liu

**Affiliations:** 1 The Ultrasound Department of Shanxi Provincial People Hospital, Shanxi Medical University, Taiyuan, Shanxi, China; 2 Department of Ultrasound, First Hospital of Shanxi Medical University, Taiyuan, Shanxi, China; 3 Department of Interventional Ultrasound, First Hospital of Shanxi Medical University, Taiyuan, Shanxi, China; 4 Department of Interventional Ultrasound, Fifth Medical Center, Chinese PLA General Hospital, Beijing, China

**Keywords:** attenuation imaging, comparative study, controlled attenuation parameter, hepatic steatosis, liver steatosis analysis, ultrasound-guided attenuation parameter

## Abstract

**Background:**

Accurate, non-invasive quantification of hepatic steatosis is crucial for clinical management. Recently developed ultrasound-guided attenuation techniques, such as Liver Steatosis Analysis (LiSA) and the Ultrasound-Guided Attenuation Parameter (UGAP), integrate real-time B-mode imaging with quantitative measurement, potentially overcoming the limitation of the lack of real-time guidance in the traditional Controlled Attenuation Parameter (CAP). However, direct comparative evidence for their performance in clinical practice remains insufficient. This study aimed to conduct a head-to-head comparison of the diagnostic performance of LiSA and UGAP, using the widely adopted CAP as a clinical reference standard.

**Methods:**

This prospective study ultimately included 357 participants. All participants underwent LiSA, UGAP, and CAP examinations during a single visit. Operations followed standardized protocols with strict quality control (IQR <40 dB/m, IQR/M <30%). CAP-defined steatosis grades (S0-S3) served as the reference. Pearson correlation analysis was used to assess the associations of each parameter with CAP, Body mass index (BMI), etc. Diagnostic performance was evaluated using receiver operating characteristic (ROC) curve analysis, and the area under the ROC curves (AUROCs) of LiSA and UGAP were compared using the DeLong test.

**Results:**

Both LiSA and UGAP demonstrated very high technical success rates (both 99.74%), significantly higher than that of CAP (96.84%, p = 0.002). Their measurements showed strong correlations with CAP values (LiSA: r = 0.83; UGAP: r = 0.81, both p < 0.001). For discriminating different steatosis grades (≥S1, ≥S2, =S3), LiSA achieved AUROCs of 0.96, 0.91, and 0.86, respectively, while UGAP achieved AUROCs of 0.96, 0.91, and 0.85, respectively. The DeLong test indicated no statistically significant difference in diagnostic performance between the two techniques across all grades (all p-values >0.62). This study provides, for the first time, CAP-referenced cut-off value suggestions for LiSA in diagnosing S2 and S3 steatosis (269.00 dB/m and 300.00 dB/m, respectively).

**Conclusion:**

LiSA and UGAP show high agreement with CAP in diagnosing and grading hepatic steatosis, with comparable performance to each other. By virtue of their inherent integration with real-time B-mode imaging, they offer higher technical success rates and operational ease, demonstrating superior clinical feasibility compared to CAP. These findings support LiSA and UGAP as effective and practical alternatives or complements to CAP, enriching the toolkit for point-of-care steatosis assessment.

## 1 Introduction

Hepatic steatosis, defined as the pathological accumulation of fat exceeding 5% of liver weight, is the most common chronic liver disease globally, affecting approximately one-quarter of adults and posing a significant risk for metabolic comorbidities, hepatocellular carcinoma, and progressive liver disease (Israelsen et al., 2024; Younossi et al., 2023; Paik et al., 2023). Accurate detection and quantification of hepatic fat are therefore critical for clinical management and prognosis.

Although liver biopsy remains the histological gold standard, its invasiveness, sampling variability, and cost limit its utility for routine screening and monitoring (Ratziu et al., 2005; Rinella and Sanyal, 2016). Non-invasive imaging alternatives have thus been sought. Magnetic resonance imaging-proton density fat fraction (MRI-PDFF) is highly accurate but is constrained by cost, availability, and patient tolerability (Azizi et al., 2025; Al-Huneidi et al., 2025). Conventional B-mode ultrasound, while widely available, lacks reliable quantitative capability for grading (Dasarathy et al., 2009). The controlled attenuation parameter (CAP), integrated with vibration-controlled transient elastography (FibroScan®), has emerged as a validated point-of-care tool for steatosis assessment (Petroff et al., 2021; Karlas et al., 2017). However, CAP lacks real-time B-mode imaging guidance, which can affect measurement success, particularly in obese patients.

To overcome these limitations, several ultrasound manufacturers have recently developed novel attenuation-based techniques that integrate quantitative attenuation measurement with real-time B-mode imaging, enabling simultaneous anatomical visualization and fat quantification (Ferraioli et al., 2022; Wu et al., 2025). Among these are the ultrasound-guided attenuation parameter (UGAP, GE Healthcare) and liver steatosis analysis (LiSA, Mindray) (Dillman et al., 2022; Imajo et al., 2022; Ren et al., 2021; Ren et al., 2022). Recent international guidelines from the World Federation for Ultrasound in Medicine and Biology (WFUMB) and the joint EASL–EASD–EASO clinical practice guidelines endorse the use of such quantitative ultrasound methods for assessing metabolic dysfunction-associated steatotic liver disease (MASLD) (Ferraioli et al., 2024; EASL-EASD-EASO, 2024). However, while initial validation studies for each technique have been published, a direct head-to-head comparison of their diagnostic performance is lacking. Such a comparison is essential to inform clinical adoption and guideline implementation.

Therefore, the aim of this study was to perform a direct comparative evaluation of the diagnostic performance of LiSA and UGAP in detecting and grading hepatic steatosis, using the clinically practical and widely adopted CAP as the reference standard.

## 2 Materials and methods

### 2.1 Ethical statement

This study was conducted in accordance with the Declaration of Helsinki. The Ethics Committee of the First Hospital of Shanxi Medical University approved the protocol (Reference No. KYLL-2023–132). All participants provided written informed consent before enrollment.

### 2.2 Participants

This prospective study enrolled 380 consecutive adults (≥18 years) diagnosed with or suspected of having fatty liver disease between September 2022 and March 2023 at the First Affiliated Hospital of Shanxi Medical University. Participants completed three imaging assessments: LiSA, UGAP, and CAP.

Exclusion criteria: age <18 years; pregnancy; history of liver surgery; presence of focal liver lesions >5 cm or ascites; serum ALT or AST >5× upper limit of normal. The final analysis included 357 participants after applying exclusion criteria and excluding cases with measurement failure or suboptimal image quality.

### 2.3 Imaging examination protocol

All examinations were performed by two experienced sonographers (each with >5 years of abdominal ultrasound experience) during a single visit. Both operators completed standardized manufacturer-provided training on LiSA and UGAP measurements. Operator consistency was validated in a pre-trial cohort (n ≥ 100; see Supplementary Material 1). Participants fasted >4 h, abstained from strenuous activity for 24 h, and rested seated for 20 min before the exam. Height and body weight were measured by a trained nurse using calibrated equipment. Body mass index (BMI) was calculated as weight in kilograms divided by the square of height in meters (kg/m^2^).

#### 2.3.1 B-mode ultrasound localization

**FIGURE 1:**
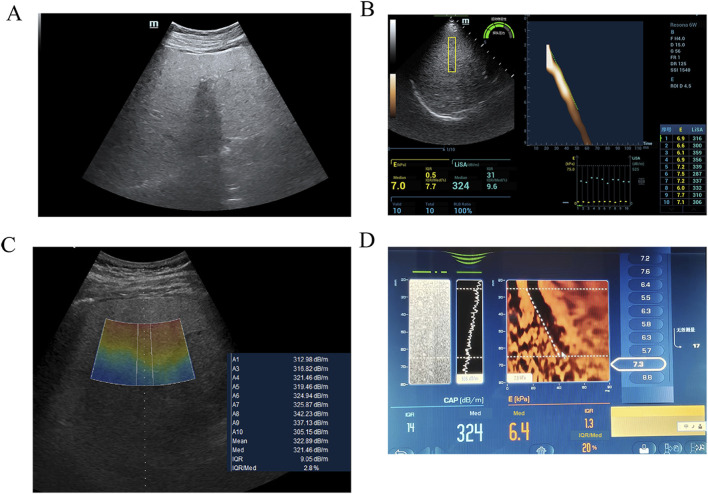
Imaging findings of severe hepatic steatosis in a 60 year-old female with hypertension. **(A)** B-mode ultrasound examination indicating severe hepatic steatosis. **(B)** UGAP measurement by GE LOGIQ E11, the value was 321 dB/m **(C)** LiSA measurement by Mindray Resona 6w, the value was 324 dB/m **(D)** CAP measurement by FibroScan, the value was 335 dB/m.

Conventional B-mode ultrasound (Mindray Resona 6w or GE LOGIQ E11) was performed to confirm diffuse hepatic steatosis (increased echogenicity, beam attenuation) and exclude focal lesions or ascites (Figure 1A). A standardized acoustic window in segment V of the right hepatic lobe was selected—homogeneous and free of major vessels or ducts. Skin-to-capsule distance (SCD) was measured from the skin surface to Glisson’s capsule using electronic calipers.

#### 2.3.2 LiSA and UGAP measurement

Under real-time B-mode guidance, the probe was placed perpendicular to the skin in the intercostal space. Participants held their breath at the end of normal expiration. Ten consecutive measurements were obtained per modality. A measurement was considered valid if the interquartile range (IQR) was <40 dB/m and the IQR-to-median ratio (IQR/M) was <30%. The final value was the median of ten valid measurements (unit: dB/m).

LiSA measurements were performed using a Resona 6w system (Mindray) equipped with an LFP5-1U convex array probe, operating in harmonic imaging mode at a frequency of 4.0 MHz. A rectangular sampling box (4.0 cm × 1.0 cm) was placed at a fixed depth of 4.5 cm (Figure 1B).

UGAP measurements were obtained using a LOGIQ E11 system (GE Healthcare) with a C1-6-D convex array probe, operating in fundamental imaging mode at 3.5 MHz. A trapezoidal sampling region (upper base 1.0 cm, lower base 1.2 cm, height 4.0 cm) was positioned within a depth range of 4–8 cm (Figure 1C).

Detailed acquisition parameters are summarized in Supplementary Table S1.

#### 2.3.3 CAP measurement

CAP was measured using a FibroScan® 502 Touch device (Echosens, France) with an M-type probe. For participants with a SCD ≥2.5 cm, the XL probe is recommended; however, in this study, the M probe was used uniformly to maintain consistent measurement conditions. The measurement site was pre-localized with a handheld ultrasound device (VScan, GE Healthcare) to avoid major ducts. The M probe was placed perpendicular to the skin; measurements started when the probe indicator turned green. At least ten valid measurements (IQR <40 dB/m, IQR/M < 30%) were obtained. The final CAP value was the median of all valid measurements (dB/m) (Figure 1D). Steatosis was graded per manufacturer-recommended thresholds: S0 (<230 dB/m), S1 (230–274 dB/m), S2 (275–299 dB/m), S3 (≥300 dB/m). Although these thresholds are widely accepted clinically, their applicability may vary across different populations. Local validation is recommended when applying these cut-offs in clinical practice.

### 2.4 Statistical analysis

Data were analyzed using SPSS 26.0 (IBM) and MedCalc 20.100. Normality was assessed with the Kolmogorov–Smirnov test. Normally distributed data are presented as mean ± standard deviation (SD); non-normal data as median (interquartile range, IQR). Categorical variables are presented as frequencies (percentages). Between-group comparisons for LiSA and UGAP across steatosis grades were performed using the Kruskal–Wallis test, followed by Dunn’s post-hoc test.

All variables involved in correlation analysis (LiSA, UGAP, CAP, BMI, SCD, age) were normally distributed in the overall cohort. Pearson’s correlation coefficient (r) was used to assess associations; correlation strength was interpreted as: r < 0.2 (negligible), 0.2–0.4 (weak), 0.4–0.7 (moderate), ≥0.7 (strong).

Diagnostic performance was evaluated using receiver operating characteristic (ROC) curve analysis. Areas under the ROC curves (AUROCs) were compared with the DeLong test. Optimal cut-off values were determined using the Youden index. A two-sided p < 0.05 was considered statistically significant.

## 3 Results

### 3.1 Patient selection and characteristics

A total of 380 patients were initially enrolled and underwent attempted measurements with LiSA, UGAP, and CAP. One patient with concurrent viral hepatitis and significantly reduced liver volume failed to yield valid measurements with any of the three modalities. An additional 11 patients failed the CAP measurement; the average BMI of this subgroup was 37.2 kg/m^2^, suggesting that obesity might be a significant contributing factor to the higher rate of CAP measurement failure. Thus, the technical success rates for LiSA, UGAP, and CAP were 99.74% (379/380; 95% CI: 98.6%–100.0%), 99.74% (379/380; 95% CI: 98.6%–100.0%), and 96.84% (368/380; 95% CI: 94.7%–98.4%), respectively. Pairwise comparisons using Fisher’s exact test showed no significant difference between the success rates of LiSA and UGAP (p = 1.000), whereas both LiSA and UGAP demonstrated significantly higher success rates than CAP (both p = 0.002). Subsequently, a further 11 patients were excluded due to suboptimal B-mode image quality, which precluded reliable quantitative analysis. Therefore, data from 357 patients were included in the final comparative and diagnostic performance analysis. The baseline characteristics of these 357 patients are presented in Table 1.

**TABLE 1:** Characteristics of enrolled patients.

Items	Summary statistics
Sex (male/female)	51.8%/48.2%
Age (years)	46.73 ± 13.25
BMI (kg/m^2^)	25.14 ± 3.87
SCD (cm)	1.84 ± 0.39

BMI, body mass index; SCD, skin-to-capsule distance.

Data are expressed as the mean ± standard deviation for continuous variables.

### 3.2 Correlations of LiSA and UGAP measurements with clinical parameters

Given the normal distribution of all variables, Pearson correlation analysis revealed strong correlations between both LiSA and UGAP values and CAP measurements (LiSA: r = 0.83, *p* < 0.001; UGAP: r = 0.81, *p* < 0.001), BMI (LiSA: r = 0.48, *p* < 0.001; UGAP: r = 0.45, *p* < 0.001), and SCD (LiSA: r = 0.45, *p* < 0.001; UGAP: r = 0.44, *p* < 0.001). No significant correlation was identified with age (LiSA: *p* = 0.34; UGAP: *p* = 0.58). These findings are presented in Supplementary Figure S1 and Supplementary Table S2.

### 3.3 Diagnostic performance of LiSA and UGAP using CAP as the comparative standard

**FIGURE 2:**
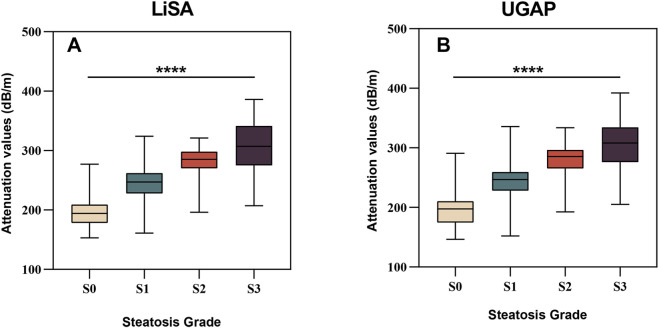
Distribution of LiSA and UGAP values across different hepatic steatosis grades. **(A)** LiSA values (dB/m) for S0 to S3 grades. **(B)** UGAP values (dB/m) for S0 to S3 grades. Both LiSA and UGAP values increased significantly with steatosis severity (p < 0.001, Kruskal‒Wallis test).

Patients were categorized into four steatosis grades (S0–S3) according to CAP values. Both LiSA and UGAP values increased significantly with steatosis severity (p < 0.001, Kruskal–Wallis test), as detailed in Table 2 and illustrated in Figure 2.

**TABLE 2:** LiSA and UGAP values of different hepatic steatosis grades using CAP as a reference.

Parameter	Hepatic steatosis grade using CAP as reference				P value
	S0	S1	S2	S3	
Number (percentage)	71 (19.89%)	118 (33.05%)	87 (24.37%)	81 (22.69%)	-
LiSA (dB/m)	194.00 (178.50–209.00)	247.00 (227.75–262.00)	285.00 (270.00–298.00)	307.00 (275.00–341.50)	<0.001
UGAP (dB/m)	197.51 (174.87–210.50)	246.73 (228.25–259.50)	285.46 (265.35–296.39)	308.31 (276.27–334.45)	<0.001

LiSA, liver steatosis analysis; UGAP, ultrasound-guided attenuation parameter.

Data are presented as median (interquartile range). Between-group comparisons were performed using the Kruskal–Wallis H test, followed by Dunn’s post-hoc test for pairwise comparisons if significant.

**FIGURE 3:**
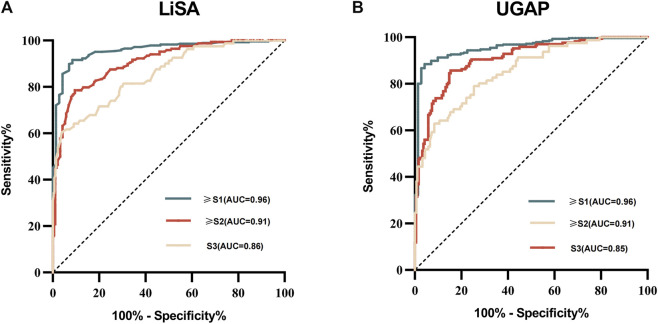
ROC curves of the LiSA and UGAP for assessing different stages of hepatic steatosis. **(A)** ROC curves for LiSA in discriminating steatosis grades ≥ S1 (AUC = 0.96), ≥S2 (AUC = 0.91), and S3 (AUC = 0.86). **(B)** ROC curves for UGAP in discriminating steatosis grades ≥ S1 (AUC = 0.96), ≥S2 (AUC = 0.91), and S3 (AUC = 0.85).

ROC curves for LiSA and UGAP are presented in Figure 3. The detailed diagnostic performance metrics for both methods are presented in Table 3.

**TABLE 3:** Optimal cut-off values of LiSA and UGAP in prediction of grades of hepatic steatosis.

Steatosis stage	Prevalence	Cut-off value (dB/m)	AUROC (95% CI)	Sensitivity (%)	Specificity (%)
LiSA					
S ≥ 1	286 (80.11%)	220.00	0.96 (0.93–0.98)	91.6	91.6
S ≥ 2	168 (47.06%)	269.00	0.91 (0.87–0.94)	78.6	90.5
S = 3	81 (22.69%)	300.00	0.86 (0.82–0.89)	60.5	96.0
UGAP					
S ≥ 1	286 (80.11%)	226.75	0.96 (0.93–0.97)	88.5	95.8
S ≥ 2	168 (47.06%)	260.00	0.91 (0.87–0.93)	85.7	84.7
S = 3	81 (22.69%)	297.18	0.85 (0.81–0.89)	63.0	91.7

AUROC, area under the receiver-operating characteristic curve; CI, confidence interval; LiSA, liver steatosis analysis; UGAP, ultrasound-guided attenuation parameter; PPV, positive predictive value; NPV, negative predictive value.

For mild hepatic steatosis (grade ≥1), LiSA achieved an AUROC of 0.96 [95% CI, 0.93–0.98] with an optimal threshold of 220.00 dB/m, resulting in both sensitivity and specificity of 91.6%. In moderate steatosis (grade ≥2), LiSA demonstrated an AUROC of 0.91 [95% CI, 0.87–0.94] at a threshold of 269.00 dB/m, yielding a sensitivity of 78.6% and specificity of 90.5%. In the detection of severe steatosis (grade = 3), the AUROC for LiSA declined to 0.86 [95% CI, 0.82–0.89], with a threshold of 300.00 dB/m, sensitivity of 60.5%, and specificity of 96.0%.

UGAP also demonstrated high diagnostic performance. For mild steatosis, the AUROC was 0.96 [95% CI, 0.93–0.97] with a cut-off of 226.75 dB/m, sensitivity of 88.5%, and specificity of 95.8%. In moderate steatosis (grade ≥2), the AUROC was 0.91 [95% CI, 0.87–0.93] at a threshold of 260.00 dB/m, with a sensitivity of 85.7% and specificity of 84.7%. For severe steatosis (grade = 3), UGAP yielded an AUROC of 0.85 [95% CI, 0.81–0.89], with a cut-off of 297.18 dB/m, sensitivity of 63.0%, and specificity of 91.7%.

### 3.4 Comparison of AUROC for different grades of hepatic steatosis: LiSA vs. UGAP

Although both LiSA and UGAP achieved AUROC values greater than 0.8 across all steatosis grades, DeLong’s test revealed no statistically significant differences in diagnostic performance between the two methods for any steatosis threshold (≥1, ≥2, or = 3). The corresponding *p* values were 0.62, 0.90, and 0.93, respectively, as presented in Table 4.

**TABLE 4:** Comparison of AUROCs of LiSA and UGAP.

Steatosis stage	AUROC of LiSA	AUROC of UGAP	Z value	P
S ≥ 1	0.96	0.96	0.49	0.62
S ≥ 2	0.91	0.91	0.13	0.90
S = 3	0.86	0.85	0.09	0.93

AUROC, area under the receiver-operating characteristic curve; LiSA, liver steatosis analysis; UGAP, ultrasound-guided attenuation parameter.

## 4 Discussion

This study aimed to conduct a direct, head-to-head comparison of the diagnostic performance of two recently developed ultrasound-based attenuation parameters—Liver Steatosis Analysis (LiSA) and the Ultrasound-Guided Attenuation Parameter (UGAP)—in the detection and grading of hepatic steatosis. We selected the Controlled Attenuation Parameter (CAP) as the pragmatic clinical reference standard for this comparison. This choice was based on CAP’s status as a widely histologically validated technology, whose diagnostic efficacy and reproducibility for different steatosis grades have been confirmed in multiple studies (Petroff et al., 2021; Karlas et al., 2017). Although liver biopsy remains the histological gold standard and magnetic resonance imaging-proton density fat fraction (MRI-PDFF) is regarded as the ideal non-invasive reference (Azizi et al., 2025; Castera et al., 2019), the adoption of these standards was constrained in this large-scale, multi-technique parallel comparison study. The invasiveness of biopsy and the cost, accessibility, and patient burden associated with MRI-PDFF made CAP a suitable benchmark that balances clinical relevance, feasibility, and efficiency. We fully acknowledge the inherent methodological considerations of using one attenuation-based technique (CAP) to evaluate other attenuation-based methods. Recent international guidelines (e.g., from the WFUMB) recommend prioritizing MRI-PDFF as the reference standard in diagnostic accuracy studies to circumvent the potential bias of “circular comparison” (Ferraioli et al., 2024). Consequently, the results of this study primarily elucidate the concordance of LiSA and UGAP with the current mainstream clinical tool (CAP), providing evidence for their integration into the existing diagnostic and management framework, rather than validating their absolute accuracy in fat quantification.

Our findings demonstrate that both LiSA and UGAP exhibit strong diagnostic agreement with CAP, and show no statistically significant difference in performance from each other, supporting their potential as effective non-invasive tools for hepatic steatosis assessment. It is noteworthy that CAP has a key practical limitation: the lack of real-time B-mode imaging guidance. This may lead to challenges in probe positioning, particularly in obese patients, thereby affecting measurement success rates (Wong, 2013). De Lédinghen et al. reported a CAP success rate of approximately 92.3% in a large cohort (de Lédinghen et al., 2014). In our study, despite using a handheld ultrasound device for pre-localization, the success rate of CAP (96.84%) remained significantly lower than that of both LiSA and UGAP (both 99.74%). This contrast highlights the core operational advantage of LiSA and UGAP: their inherent integration with real-time B-mode imaging. This image-guidance capability allows operators to intuitively select sampling regions and avoid major structures, thereby greatly enhancing measurement reproducibility, success rate, and operator confidence, consistent with previous reports on the high feasibility of such techniques (Ren et al., 2021; Bende et al., 2021).

The primary quantitative evidence supporting their clinical validity is the strong correlation observed between the measurements of both LiSA and UGAP with CAP values (LiSA: r = 0.83; UGAP: r = 0.81), aligning with the trend reported by Fujiwara et al. for UGAP (r = 0.73) (Fujiwara et al., 2018). Both parameters also showed significant positive correlations with BMI and SCD, in accordance with the physical principles of ultrasound attenuation and consistent with other studies (Ren et al., 2021), further validating their reliability in reflecting physiological changes associated with fat deposition.

Regarding diagnostic performance, both UGAP and LiSA demonstrated excellent discriminatory ability for grading hepatic steatosis in our cohort, with areas under the receiver operating characteristic curves (AUROCs) exceeding 0.85 for all severity grades (≥S1, ≥S2, =S3). The performance of UGAP (AUROCs: 0.96, 0.91, 0.85) was highly consistent with previous validation studies using MRI-PDFF and CT as references (Imajo et al., 2022; Iwashita et al., 2022). Although Imajo et al. reported a slightly higher AUROC for S3 (0.89) using MRI-PDFF as reference (Imajo et al., 2022), the minor discrepancy may be attributed to characteristics of the reference standard itself or population differences. Importantly, the conclusions drawn from our study using CAP as a reference in a Chinese population are corroborated by studies employing different reference standards (MRI-PDFF, CT) and other CAP-referenced validations in different populations (Japanese, European) (Imajo et al., 2022; Bende et al., 2021; Iwashita et al., 2022), collectively strengthening the evidence for UGAP’s diagnostic robustness and its applicability across clinical and geographical settings.

Similarly, LiSA demonstrated comparable diagnostic efficacy (AUROCs: 0.96, 0.91, 0.86). Although direct literature on LiSA is more limited, its performance aligns with the overall trends observed for UGAP and other attenuation-based techniques. The numerically slightly lower diagnostic performance metrics for LiSA reported by Ren et al. against liver biopsy (Ren et al., 2021; Ren et al., 2022) likely reflect the more stringent diagnostic threshold of histology as a gold standard, yet they still affirm LiSA’s fundamental capability to discriminate clinically relevant steatosis thresholds. The convergence of high diagnostic accuracy between these two independently developed technologies—despite potential differences in technical implementations such as imaging mode and region-of-interest geometry—underscores the maturity and reliability of ultrasound-based attenuation measurement as a methodological principle for fat quantification.

The consistent diagnostic accuracy necessitates the determination of reliable thresholds for clinical decision-making. The CAP-referenced cut-off values for UGAP proposed in this study (226.75, 260.00, 297.18 dB/m) are close to, yet show a consistent, slightly higher trend compared to the MRI-PDFF-referenced values (227.5, 248.5, 269.5 dB/m) reported by Imajo et al. (2022). This systematic shift may reflect inherent calibration differences between CAP and MRI-PDFF, or suggest population-specific influences, a phenomenon also noted in cross-study comparisons of CAP-referenced UGAP cut-offs (Huang et al., 2025; Cannella et al., 2025). This clearly indicates that cut-off values are highly dependent on the reference standard and specific population, and are not universal constants. For LiSA, this study provides, for the first time, CAP-referenced diagnostic threshold suggestions for moderate and severe steatosis (269.00 and 300.00 dB/m), addressing an evidence gap (Ren et al., 2022). Its mild steatosis threshold (220.00 dB/m) aligns directionally with Ren et al.'s biopsy-based threshold for ≥5% fat (236.00 dB/m), providing valuable cross-reference (Ren et al., 2022). These findings emphasize the crucial need for local validation or careful contextual assessment when applying specific thresholds of any technique in clinical practice.

A noteworthy finding in our results is the relatively lower sensitivity of both LiSA and UGAP for detecting severe steatosis (S3, ∼60–63%) compared to their ability to detect milder grades. This phenomenon requires interpretation in the context of multiple factors. First, the S3 group as defined by CAP itself exhibited the greatest dispersion in measurement values, suggesting significant heterogeneity within this group—a “spectrum effect” known to inherently increase the difficulty of precise classification at disease extremes. Second, the reference standard CAP itself does not have 100% sensitivity (∼88%) (Karlas et al., 2017) for histologically diagnosed S3, implying that some cases categorized as S3 by CAP may lie near its diagnostic threshold. Therefore, discrepancies between LiSA/UGAP and CAP in this zone may partially reflect legitimate inter-technique variance rather than failure of the new technologies. Additionally, potential signal dynamics at very high fat concentrations, and the measurement complexity introduced by the higher BMI and metabolic abnormalities commonly associated with severe steatosis, may also influence inter-technique agreement (de Lédinghen et al., 2014). Consequently, for assessing severe steatosis near diagnostic thresholds, emphasis should be placed on comprehensive judgment incorporating clinical information and other indicators.

The high technical success rates of LiSA and UGAP lay a solid operational foundation for their clinical adoption. On this premise, the direct comparison of their diagnostic performance becomes central. DeLong’s test confirmed no statistically significant difference in the diagnostic performance between the two across all steatosis grades (p ≥ 0.62). Most importantly, this diagnostic equivalence is strongly supported by their excellent methodological consistency. An independent analysis based on the same cohort confirmed both excellent intra- and inter-observer agreement (ICCs: 0.97–0.99) and excellent inter-platform agreement between LiSA and UGAP (ICC: 0.96). Bland-Altman analysis further demonstrated minimal bias (mean difference: 0.12 dB/m) with clinically acceptable limits of agreement (95% LOAs: −29.08–29.32 dB/m) (Chen et al., 2025). Therefore, this diagnostic equivalence, combined with high methodological consistency and their strong correlation with CAP, collectively supports a key conclusion: within the frame of reference of the current mainstream CAP evaluation system, these two image-guided attenuation techniques possess dual clinical and methodological interchangeability for steatosis grading. This finding has direct practical implications: it suggests that when introducing such technologies into clinical practice, factors such as device platform availability, integration efficiency with existing workflows, operator training, and cost-effectiveness may become more important decision-making factors than subtle differences in diagnostic performance. Furthermore, the consistency and equivalent performance between the two technologies, as well as between them and CAP, collectively consolidate the body of evidence for “image-guided ultrasound attenuation measurement” as a reliable class of technology, potentially aiding future clinical guidelines in endorsing this class of validated technological methods rather than specific proprietary implementations.

This study has several limitations. First, it is a single-center study with a relatively homogeneous population (Chinese, mean BMI 25.14 kg/m^2^), which limits the generalizability of the findings and the proposed diagnostic thresholds to other ethnicities, individuals with extreme obesity, or those with coexisting chronic liver diseases of other etiologies. Second, the two more precise standards, liver biopsy and MRI-PDFF, were not included in the current analysis. The use of CAP as a reference standard was a pragmatic choice based on clinical reality, but its inherent “attenuation vs. attenuation” comparison paradigm means this study primarily validates consistency with existing clinical practice. Future studies designed for direct comparison with MRI-PDFF or biopsy are warranted to independently verify the absolute quantitative accuracy of these techniques. Additionally, this cross-sectional study did not assess the value of LiSA and UGAP in longitudinally monitoring disease progression or treatment response, which is an important next step in defining their comprehensive clinical utility.

The methodology and findings of this study are consistent with the spirit of recent international guidelines. The WFUMB guidelines on liver fat quantification support the use of standardized ultrasound attenuation techniques and emphasize operational standardization (Ferraioli et al., 2024). The joint EASL–EASD–EASO guidelines recognize the important role of non-invasive tools including CAP in the assessment and management of metabolic dysfunction-associated steatotic liver disease (MASLD) (EASL-EASD-EASO, 2024). Our work, through direct comparison, adds specific evidence to such guideline recommendations regarding the excellent and equivalent performance of emerging image-guided techniques and clarifies their operational advantages over traditional methods.

## 5 Conclusion

In conclusion, both LiSA and UGAP demonstrate strong agreement with CAP in quantifying hepatic steatosis, with no statistically significant difference in their diagnostic performance. Their high technical success rates and operational ease underscore superior clinical feasibility for integration into routine ultrasound practice. These findings position LiSA and UGAP as comparable and practical alternatives to CAP, enhancing the toolkit for point-of-care steatosis assessment.

## Data Availability

The raw data supporting the conclusions of this article will be made available by the authors, without undue reservation.

## References

[B1] Al-Huneidi L. I. Zhao F. Maas R. Ermans S. J. E. Runge J. Chen X. (2025). Liver fat quantification and steatosis grading in fatty liver disease by magnetic resonance imaging: systematic review and meta-analysis. J. Gastroenterol. Hepatol. 40, 2808–2819. 10.1111/jgh.70086 41117160 PMC12666615

[B2] Azizi N. Naghibi H. Shakiba M. Morsali M. Zarei D. Abbastabar H. (2025). Evaluation of MRI proton density fat fraction in hepatic steatosis: a systematic review and meta-analysis. Eur. Radiol. 35, 1794–1807. 10.1007/s00330-024-11001-1 39254718

[B3] Bende F. Sporea I. Șirli R. Bâldea V. Lazăr A. Lupuşoru R. (2021). Ultrasound-guided attenuation parameter (UGAP) for the quantification of liver steatosis using the controlled attenuation parameter (CAP) as the reference method. Med. Ultrason. 23, 7–14. 10.11152/mu-2688 33220028

[B4] Cannella R. Agnello F. Porrello G. Spinello A. U. Infantino G. Pennisi G. (2025). Performance of ultrasound-guided attenuation parameter and 2D shear wave elastography in patients with metabolic dysfunction-associated steatotic liver disease. Eur. Radiol. 35, 2339–2350. 10.1007/s00330-024-11076-w 39373742 PMC11914239

[B5] Castera L. Friedrich-Rust M. Loomba R. (2019). Noninvasive assessment of liver disease in patients with nonalcoholic fatty liver disease. Gastroenterology 156, 1264–1281.e4. 10.1053/j.gastro.2018.12.036 30660725 PMC7505052

[B6] Chen J. Hao Y. H. Zhang Y. J. Liu J. J. Liang P. Liu L. P. (2025). Interplatform agreement between liver steatosis analysis and ultrasound-guided attenuation parameter in the evaluation of hepatic steatosis. J. Multidiscip. Healthc. 18, 4023–4032. 10.2147/JMDH.S528289 40687267 PMC12275988

[B7] Dasarathy S. Dasarathy J. Khiyami A. Joseph R. Lopez R. McCullough A. J. (2009). Validity of real time ultrasound in the diagnosis of hepatic steatosis: a prospective study. J. Hepatol. 51, 1061–1067. 10.1016/j.jhep.2009.09.001 19846234 PMC6136148

[B8] de Lédinghen V. Vergniol J. Capdepont M. Chermak F. Hiriart J. B. Cassinotto C. (2014). Controlled attenuation parameter (CAP) for the diagnosis of steatosis: a prospective study of 5323 examinations. J. Hepatol. 60, 1026–1031. 10.1016/j.jhep.2013.12.018 24378529

[B9] Dillman J. R. Thapaliya S. Tkach J. A. Trout A. T. (2022). Quantification of hepatic steatosis by ultrasound: prospective comparison with MRI proton density fat fraction as reference standard. AJR Am. J. Roentgenol. 219, 784–791. 10.2214/AJR.22.27878 35674351

[B10] EASL-EASD-EASO (2024). European Association for the Study of the Liver (EASL); European Association for the Study of Diabetes (EASD); European Association for the Study of Obesity (EASO). EASL-EASD-EASO Clinical Practice Guidelines on the management of metabolic dysfunction-associated steatotic liver disease (MASLD). J. Hepatol. 81 492–542. 10.1016/j.jhep.2024.04.031 38851997

[B11] Ferraioli G. Kumar V. Ozturk A. Nam K. de Korte C. L. Barr R. G. (2022). US attenuation for liver fat quantification: an AIUM-RSNA QIBA pulse-echo quantitative ultrasound initiative. Radiology 302, 495–506. 10.1148/radiol.210736 35076304

[B12] Ferraioli G. Barr R. G. Berzigotti A. Sporea I. Wong V. W. Reiberger T. (2024). WFUMB guidelines/guidance on liver multiparametric ultrasound. Part 2: Guidance on liver fat quantification. Ultrasound Med. Biol. 50, 1088–1098. 10.1016/j.ultrasmedbio.2024.03.014 38658207

[B13] Fujiwara Y. Kuroda H. Abe T. Ishida K. Oguri T. Noguchi S. (2018). The B-Mode image-guided ultrasound attenuation parameter accurately detects hepatic steatosis in chronic liver disease. Ultrasound Med. Biol. 44, 2223–2232. 10.1016/j.ultrasmedbio.2018.06.017 30077415

[B14] Huang Y. L. Sun C. Wang Y. Cheng J. Wang S. W. Wei L. (2025). Ultrasound-guided attenuation parameter for identifying metabolic dysfunction-associated steatotic liver disease: a prospective study. Ultrasonography 44, 134–144. 10.14366/usg.24204 39935289 PMC11938800

[B15] Imajo K. Toyoda H. Yasuda S. Suzuki Y. Sugimoto K. Kuroda H. (2022). Utility of ultrasound-guided attenuation parameter for grading steatosis with reference to MRI-PDFF in a large cohort. Clin. Gastroenterol. Hepatol. 20, 2533–2541.e7. 10.1016/j.cgh.2021.11.003 34768008

[B16] Israelsen M. Francque S. Tsochatzis E. A. Krag A. (2024). Steatotic liver disease. Lancet 404, 1761–1778. 10.1016/s0140-6736(24)01811-7 39488409

[B17] Iwashita H. Shakado S. Yoshimaru N. Tanaka H. Koto F. Tanaka T. (2022). Clinical utility of ultrasound-guided attenuation parameter for the detection and quantification of hepatic steatosis in patients with fatty liver diagnosed by computed tomography. Ultrasound Med. Biol. 48, 1282–1289. 10.1016/j.ultrasmedbio.2022.02.023 35397929

[B18] Karlas T. Petroff D. Sasso M. Fan J. G. Mi Y. Q. de Lédinghen V. (2017). Individual patient data meta-analysis of controlled attenuation parameter (CAP) technology for assessing steatosis. J. Hepatol. 66, 1022–1030. 10.1016/j.jhep.2016.12.022 28039099

[B19] Paik J. M. Henry L. Younossi Y. Ong J. Alqahtani S. Younossi Z. M. (2023). The burden of nonalcoholic fatty liver disease (NAFLD) is rapidly growing in every region of the world from 1990 to 2019. Hepatol. Commun. 7, e0251. 10.1097/HC9.0000000000000251 37782469 PMC10545420

[B20] Petroff D. Blank V. Newsome P. N. Voican C. S. Thiele M. de Lédinghen V. (2021). Assessment of hepatic steatosis by controlled attenuation parameter using the M and XL probes: an individual patient data meta-analysis. Lancet Gastroenterol. Hepatol. 6, 185–198. 10.1016/s2468-1253(20)30357-5 33460567

[B21] Ratziu V. Charlotte F. Heurtier A. Gombert S. Giral P. Bruckert E. (2005). Sampling variability of liver biopsy in nonalcoholic fatty liver disease. Gastroenterology 128, 1898–1906. 10.1053/j.gastro.2005.03.084 15940625

[B22] Ren X. Xia S. Zhang L. Li R. Zhou W. Ji R. (2021). Analysis of liver steatosis analysis and controlled attenuation parameter for grading liver steatosis in patients with chronic hepatitis B. Quant. Imaging Med. Surg. 11, 571–578. 10.21037/qims-19-1091 33532257 PMC7779909

[B23] Ren X. Wang J. Xia S. Zhan W. Li R. Chen Z. (2022). A new visual quantitative assessment of ultrasound attenuation parameters for the mild liver steatosis. Ann. Transl. Med. 10, 343. 10.21037/atm-22-989 35433934 PMC9011258

[B24] Rinella M. E. Sanyal A. J. (2016). Management of NAFLD: a stage-based approach. Nat. Rev. Gastroenterol. Hepatol. 13, 196–205. 10.1038/nrgastro.2016.3 26907882

[B25] Wong G. L. (2013). Update of liver fibrosis and steatosis with transient elastography (fibroscan). Gastroenterol. Rep. (Oxf) 1, 19–26. 10.1093/gastro/got007 24759663 PMC3941434

[B26] Wu S. Pan J. Song M. Zhao Y. C. Chen W. Huang H. (2025). Performance of magnetic resonance imaging and ultrasound for identifying the different degrees of hepatic steatosis: a systematic review and meta-analysis. Acad. Radiol. 32, 6528–6540. 10.1016/j.acra.2025.03.008 40164534

[B27] Younossi Z. M. Golabi P. Paik J. M. Henry A. Van Dongen C. Henry L. (2023). The global epidemiology of nonalcoholic fatty liver disease (NAFLD) and nonalcoholic steatohepatitis (NASH): a systematic review. Hepatology 77, 1335–1347. 10.1097/HEP.0000000000000004 36626630 PMC10026948

