# Supplementary material for: Diagnostic performance of liver steatosis analysis and ultrasound-guided attenuation parameter in quantifying hepatic steatosis: a comparative evaluation using controlled attenuation parameter as reference
Source: Front Physiol. 2026 Feb 27;17:1752895. doi: 10.3389/fphys.2026.1752895 (PMC12982092; doi:10.3389/fphys.2026.1752895)
Supplement: Supplementary file 1 [file Supplementaryfile1.docx]

**Supplemental Material 1. Operator Training and Consistency Validation**

Prior to the study, both sonographers completed standardized training provided by the manufacturers (Mindray and GE Healthcare) on the acquisition and interpretation of LiSA and UGAP measurements.

To ensure measurement consistency, a pre‑trial validation was performed on an independent cohort of 120 consecutive patients with suspected hepatic steatosis. Each operator independently performed LiSA and UGAP measurements in all patients.

These results confirmed excellent reproducibility, supporting the operational consistency required for the main study.
