# Supplementary material for: Diagnostic performance of liver steatosis analysis and ultrasound-guided attenuation parameter in quantifying hepatic steatosis: a comparative evaluation using controlled attenuation parameter as reference
Source: Front Physiol. 2026 Feb 27;17:1752895. doi: 10.3389/fphys.2026.1752895 (PMC12982092; doi:10.3389/fphys.2026.1752895)
Supplement: Supplementary file 2 [file Table1.docx]

**Supplemental Table 1. Detailed Acquisition Parameters for LiSA and UGAP**

| Parameter | LiSA (Mindray Resona 6w) | UGAP (GE LOGIQ E11) |
| --- | --- | --- |
| **Probe** | LFP5‑1U phased array probe | C1‑6‑D convex array |
| **Imaging mode** | Harmonic | Fundamental |
| **Frequency** | 4.0 MHz | 3.5 MHz |
| **Sampling box geometry** | Rectangle | Trapezoid |
| **Sampling box dimensions** | 4.0 cm × 1.0 cm | Upper base 1.0 cm, lower base 1.2 cm, height 4.0 cm |
| **Depth range** | Fixed at 4.5 cm | 4–8 cm |
| **Stability indicator** | On‑screen pressure & stability indicator (green) | On‑screen stability indicator |
