# Supplementary figures and images for: Diagnostic performance of liver steatosis analysis and ultrasound-guided attenuation parameter in quantifying hepatic steatosis: a comparative evaluation using controlled attenuation parameter as reference
Source: Front Physiol. 2026 Feb 27;17:1752895. doi: 10.3389/fphys.2026.1752895 (PMC12982092; doi:10.3389/fphys.2026.1752895)

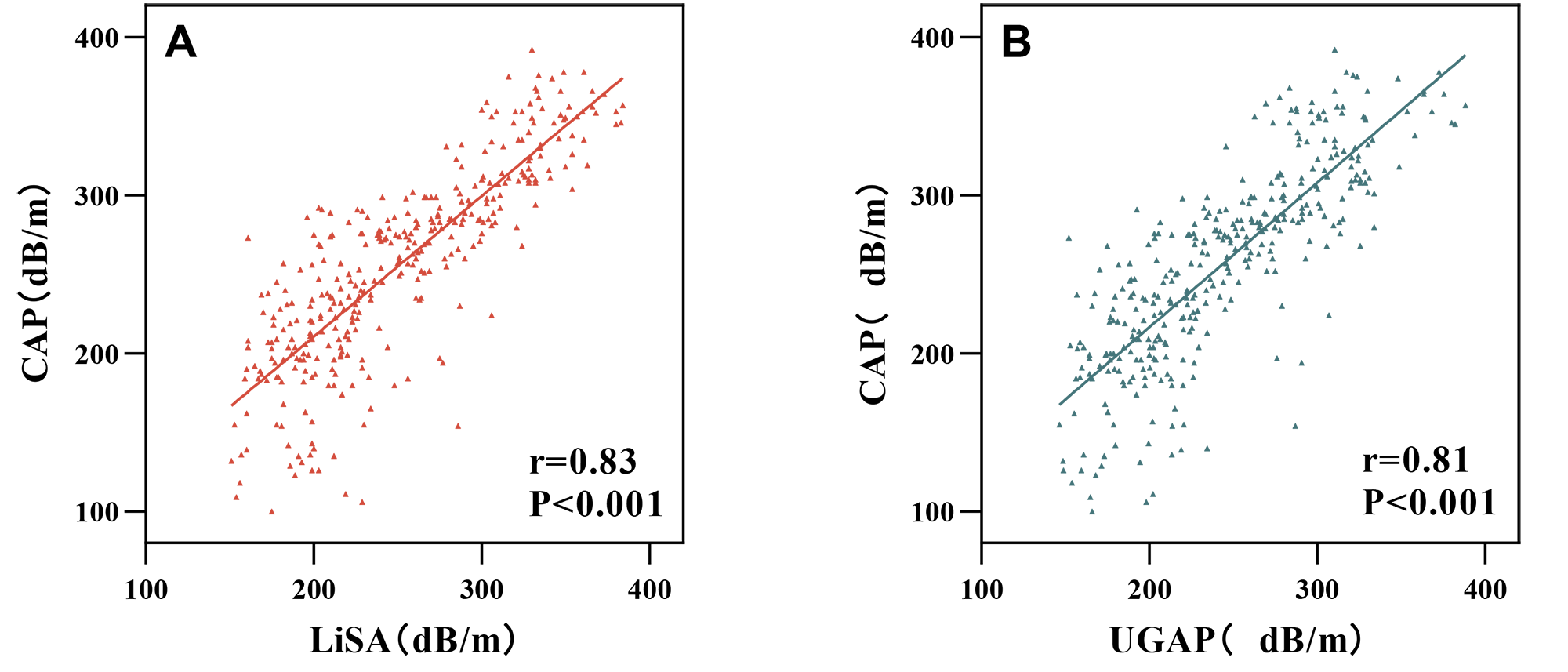

Supplement: Supplementary file 3 [file Image1.tif]
