# Supplementary material for: Diagnostic performance of liver steatosis analysis and ultrasound-guided attenuation parameter in quantifying hepatic steatosis: a comparative evaluation using controlled attenuation parameter as reference
Source: Front Physiol. 2026 Feb 27;17:1752895. doi: 10.3389/fphys.2026.1752895 (PMC12982092; doi:10.3389/fphys.2026.1752895)
Supplement: Supplementary file 4 [file Table2.docx]

**Supplemental Table 2 Correlations of LiSA and UGAP Measurements with Clinical Parameters**

|  | LiSA | | UGAP | |
| --- | --- | --- | --- | --- |
|  | r | P | r | P |
| CAP | 0.83 | <0.001^*^ | 0.81 | <0.001^*^ |
| Age | -0.51 | 0.34 | -0.03 | 0.58 |
| BMI | 0.48 | <0.001^*^ | 0.45 | <0.001^*^ |
| SCD | 0.45 | <0.001^*^ | 0.44 | <0.001^*^ |

LiSA: liver steatosis analysis; UGAP: ultrasound-guided attenuation parameter; CAP: controlled attenuation parameter; BMI: body mass index; SCD: skin-capsule distance.

^*^P<0.05
